# Substrate Use of *Pseudovibrio* sp. Growing in Ultra-Oligotrophic Seawater

**DOI:** 10.1371/journal.pone.0121675

**Published:** 2015-03-31

**Authors:** Anne Schwedt, Michael Seidel, Thorsten Dittmar, Meinhard Simon, Vladimir Bondarev, Stefano Romano, Gaute Lavik, Heide N. Schulz-Vogt

**Affiliations:** 1 Max Planck Institute for Marine Microbiology, Bremen, Germany; 2 Research Group for Marine Geochemistry (ICBM-MPI Bridging Group) Institute for Chemistry and Biology of the Marine Environment (ICBM), Carl von Ossietzky University of Oldenburg, Oldenburg, Germany; 3 Institute for Chemistry and Biology of the Marine Environment (ICBM), Carl von Ossietzky University of Oldenburg, Oldenburg, Germany; 4 Leibniz Institute for Baltic Sea Research Warnemünde, Rostock, Germany; Universidad Miguel Hernandez, SPAIN

## Abstract

Marine planktonic bacteria often live in habitats with extremely low concentrations of dissolved organic matter (DOM). To study the use of trace amounts of DOM by the facultatively oligotrophic *Pseudovibrio* sp. FO-BEG1, we investigated the composition of artificial and natural seawater before and after growth. We determined the concentrations of dissolved organic carbon (DOC), total dissolved nitrogen (TDN), free and hydrolysable amino acids, and the molecular composition of DOM by electrospray ionization Fourier transform ion cyclotron resonance mass spectrometry (ESI FT-ICR-MS). The DOC concentration of the artificial seawater we used for cultivation was 4.4 μmol C L^-1^, which was eight times lower compared to the natural oligotrophic seawater we used for parallel experiments (36 μmol C L ^-1^). During the three-week duration of the experiment, cell numbers increased from 40 cells mL^-1^ to 2x10^4^ cells mL ^-1^ in artificial and to 3x10^5^ cells mL ^-1^ in natural seawater. No nitrogen fixation and minor CO_2_ fixation (< 1% of cellular carbon) was observed. Our data show that in both media, amino acids were not the main substrate for growth. Instead, FT-ICR-MS analysis revealed usage of a variety of different dissolved organic molecules, belonging to a wide range of chemical compound groups, also containing nitrogen. The present study shows that marine heterotrophic bacteria are able to proliferate with even lower DOC concentrations than available in natural ultra-oligotrophic seawater, using unexpected organic compounds to fuel their energy, carbon and nitrogen requirements.

## Introduction

In open oceans, the concentration of dissolved organic matter (DOM) is typically expressed as the concentration of dissolved organic carbon (DOC) and is generally below 1 mg C L^-1^ (83 μmol C L^-1^) [1–2]. Consequently, marine planktonic bacteria are commonly exposed to very low concentrations of organic material. Furthermore, this organic material is an extremely diverse pool of different compounds, consisting of more than ten thousand types of molecules with different reactivities [3]. Previous studies suggest that large fractions of the DOM in the ocean are inert to bacterial break-down and are apparently not utilized by marine microorganisms [4–6], the fraction utilized by microorganisms is called assimilable organic carbon (AOC) [7]. Measurements of the consumption of specific substrates revealed that a large fraction of the labile organic material that is used in the upper ocean can consist of dissolved free amino acids (DFAA), dissolved combined amino acids (DCAA) and monosaccharides. These substances can account for 5 to 100% of the bacterial carbon and nitrogen demand [8–10]. In many natural systems, amino acids are only present in nanomolar concentrations even though the flux of amino acids is comparably high due to a close coupling of their release and uptake by planktonic microorganisms [8; 11–12]. Still, amino acids and monosaccharides alone cannot fully explain the growth of the bacteria in all cases, as they often represent only a small fraction of the bacterial C- and N-requirements [10–11]. The remaining C- and N-demand must be covered by other DOM compounds, which might be present at concentrations not detectable with the canonical analytical methods.

Among the different analytical techniques, electrospray ionization Fourier transform ion cyclotron resonance mass spectrometry (ESI FT-ICR-MS) represents yet the most appropriate and promising technique for investigating the DOM composition and the effect of marine bacteria on it. This technique provides accurate mass measurements with ppm or sub-ppm error providing ultra-high resolved mass spectra with thousands of accurate masses, which can be transformed into real elemental composition [13].

In order to investigate the specific spectrum of compounds consumed by bacteria during growth, a batch culture containing a single bacterial strain is an ideal experimental set-up, since it is a closed system with defined amounts of nutrients present. In such a system, it is possible to analyze compounds that are utilized during the growth of the strain. For the cultivation of marine bacteria, defined artificial seawater or natural seawater containing a diverse DOM pool can serve as a medium. It is important to point out that in these experiments, the risk of contamination especially with amino acids is a well-known problem [14] and therefore special attention on medium purity is required.

In the present study, we investigated the substrate use of the marine *Pseudovibrio* sp. strain FO-BEG1. Bacteria belonging to the genus *Pseudovibrio* are heterotrophic α-Proteobacteria distributed worldwide and they are metabolically extremely versatile [15]. Most of the ocean DOM is biologically recalcitrant and aged and only available at relatively low concentrations [1]. *Pseudovibrio* sp. strain FO-BEG1 is capable to grow under ultra-oligotrophic conditions in pure artificial seawater without the addition of organic substances and thus is ideal to compare the substrate use in artificial seawater with the one in aged natural oligotrophic seawater. In spite of special purification of the artificial seawater medium, a DOC concentration of 0.053 mg C L^-1^ (4.4 μmol C L^-1^) was measured, which is, nevertheless, extraordinarily low compared to typical concentrations in natural oligotrophic seawater (generally ≤1 mg C L^-1^ or ≤83 μmol C L^-1^ [1]). The natural oligotrophic seawater used in this study had a DOC concentration of 0.4 mg C L^-1^ (36 μmol C L^-1^). The molecular composition of the DOM before and after growth in artificial and natural seawater was analyzed by high performance liquid chromatography (HPLC) measuring free and hydrolysable amino acids, and by ESI FT-ICR-MS revealing changes on the level of individual molecular formulas. These methods provide detailed molecular insights into the usage of organic substrates utilized by the bacteria during growth under these ultra-oligotrophic conditions. Using isotope-ratio monitoring mass spectrometry the incorporation of nitrogen (^15^N_2_) and carbon (NaH^13^CO_3_) into *Pseudovibrio* sp. FO-BEG1 cells growing in pure artificial seawater medium was examined. Further, cells were also exposed to reduced nitrogen and carbon (ammonium and/or glucose) revealing whether or not cells are limited in carbon, nitrogen or both.

## Methods

### Medium preparation and bacterial strain

The basal artificial oligotrophic seawater medium (artificial seawater, medium 1) was composed of 518.5 mmol L^-1^ NaCl, 16.2 mmol L^-1^ MgCl_2_ × 6 H_2_O, 11.4 mmol L^-1^ MgSO_4_ × 2 H_2_O, 3 mmol L^-1^ CaCl_2_ × 2 H_2_O, 9.4 mmol L^-1^ KCl, 50 μl 2 mol L^-1^ NaOH, 2 mL 1 mol L^1^ NaHCO_3_, 1 mL 1 mmol L^-1^ K_2_HPO_4_ and 1 mL trace elements solution in 1 L MembraPure water (Optilab-Standard Water System, MembraPure, Bodenheim, Germany). The trace elements solution was composed of 7.6 μmol L^-1^ FeSO_4_ × 7 H_2_O, 0.97 mmol L^-1^ H_3_BO_3_, 0.5 mmol L^-1^ MnCl_2_ × 4 H_2_O, 0.8 mmol L^-1^ CoCl_2_ × 6 H_2_O, 0.1 mmol L^-1^ NiCl_2_ × 6 H_2_O, 11.7 μmol L^-1^ CuCl_2_ × 2 H_2_O, 0.5 mmol L^-1^ ZnSO_4_ × 7 H_2_O, 83.7 μmol L^-1^ Na_2_MoO_4_ × 2 H_2_O and 13 mL L^-1^ HCl (25%). All salts and solutions were analytical grade (p.a.) quality (Sigma-Aldrich/Fluka, Steinheim, Germany). Serum bottles (Wheaton 125 mL serum bottles clear, total volume of 156 mL, Wheaton, Millville, New Jersey, USA) were used for cultivation, filled completely with medium and closed with Teflon stoppers. For the preparation of clean artificial seawater medium (medium 2), all glassware, Teflon stoppers, screw caps and tubes were acid washed (0.1 mol L^-1^ HCl, Merck, Darmstadt, Germany) and rinsed with MembraPure water (Optilab-Standard Water System, MembraPure, Bodenheim, Germany) and glassware, aluminium foil and NaCl were combusted at 480°C for at least 3 hours. For the FT-ICR-MS experiment the medium (purified artificial seawater, medium 3) was purified before bottling of the medium by acidifying the medium and passing it through the same solid phase extraction adsorber used for sample preparation for FT-ICR-MS ([16] and see below). All incubations were performed without shaking at 28°C in the dark. To test the influence of fixed carbon and nitrogen sources on the growth of the bacteria glucose- and ammonium-addition experiments were performed. For these experiments the oligotrophic artificial seawater medium was not specifically purified before use (artificial seawater, medium 1). Glucose and ammonium were both added at a final concentration of 30 μmol L^-1^. Furthermore, growth on different carbon sources was tested using a Biolog plate and labeling experiments were performed to test fixation of CO_2_ and N_2_ (see below).

Natural surface seawater was collected in the South Pacific during Integrated Ocean Drilling (IODP) Expedition 329 from IODP Site U1368 (27.9°South; 137.9°West) with a bucket rinsed with seawater and stored at 4°C in a 2 L Nalgene (polyethylene) bottle rinsed with seawater. Before incubation, seawater was aged for 7 months and filtered through Acrodisc 25 mm syringe filters with a 0.2 μm GHP membrane (Pall Life Sciences, Ann Arbor, Michigan, USA) that were pre-washed with sterile MembraPure water and filled into pre-combusted serum bottles that were closed with Teflon stoppers. The natural seawater contained 36.4–39.3 μmol C L^-1^ at the beginning of the incubation (Table 1), which is 10–15 μmol C L^-1^ lower than expected for surface water in this region [1]. This low DOC content is probably a result of the aging of this water for 7 months. Inoculated cultures (triplicates per time point) and uninoculated controls were incubated without shaking at 28°C in the dark.

**Table 1:** Purified artificial seawater (medium 3; AS) and natural seawater (NS): Concentrations of dissolved organic carbon (DOC), total dissolved nitrogen (TDN), dissolved free amino acids (DFAA), dissolved combined amino acids (DCAA) and total hydrolysable dissolved amino acids (THDAA) at the different time points: before bottling: only medium; blank: medium poured into serum bottles before inoculation; t_0_: directly after inoculation; t_1_: 1 week after inoculation; t_2_: 3 weeks after inoculation. (n.d. = not determined).

**seawater**	**Time point**	**DOC** [μmol L^-1^]	**TDN** [μmol L^-1^]	**C in DFAA** [μmol L^-1^]	**N in DFAA** [μmol L^-1^]	**C in DCAA** [μmol L^-1^]	**N in DCAA** [μmol L^-1^]	**C in THDAA** [μmol L^-1^]	**N in THDAA** [μmol L^-1^]
AS	before bottling	1.5 ± 1.1	< 0.5	n.d.	n.d.	n.d.	n.d.	n.d.	n.d.
AS	blank	4.4 ± 0.2	< 0.5	n.d.	n.d.	n.d.	n.d.	n.d.	n.d.
AS	t_0_	4.3 ± 0.6	< 0.5	0.08	0.03	0.05	0.02	0.13	0.05
AS	t_1_	4.8 ± 0.3	< 0.5	0.02	0.01	0.12	0.05	0.14	0.06
AS	t_2_	4.3 ± 0.4	< 0.5	0.02	0.01	0.06	0.02	0.08	0.03
NS	t_0 inoculated_	36.4 ± 2.1	3.6 ± 1.5	n.d.	n.d.	n.d.	n.d.	0.08	0.02
NS	t_0 uninoculated_	39.3 ± 4.0	4.2 ± 2.5	n.d.	n.d.	n.d.	n.d.	n.d.	n.d.
NS	t_1 inoculated_	40.0 ± 0.9	4.8 ± 0.4	n.d.	n.d.	n.d.	n.d.	0.09	0.02
NS	t_1 uninoculated_	42.7 ± 1.7	4.6 ± 0.9	n.d.	n.d.	n.d.	n.d.	n.d.	n.d.

Media were inoculated with 100 μL of an oligotrophically pre-grown (clean artificial seawater, medium 2) culture of *Pseudovibrio* sp. FO-BEG1 (about 6×10^4^ cells mL^-1^), thus, the initial cell number after inoculation was calculated to be ca. 40 cells mL^-1^. The isolate *Pseudovibrio* sp. FO-BEG1 [15] was originally grown in co-culture with a marine *Beggiatoa* strain [17–19] and was isolated into pure culture by dilution series and subsequent streaking on plates [20]. The *Pseudovibrio* isolate was adapted to oligotrophic conditions since more than 25 transfers.

### Cell counts

Cells were stained with 1:5000 SYBR Green (SYBR Green I 10,000×, Sigma, Taufkirchen, Germany) for 20 minutes. The stained cells were filtered onto black filters (GTTP Isopore Membrane Filters 0.22 μm, Millipore, Schwalbach am Taunus, Germany) using a Bio-Dot apparatus (Bio-Rad, München, Germany). Cell counting was performed using a fluorescence microscope (Axiophot, Zeiss, Jena, Germany) at 450–490 nm excitation and 515–565 nm emission (filter set 10, Zeiss, Jena, Germany). For each sample, 10 fields were counted. All cell counts were performed for at least three biological replicates.

### Sampling and solid phase extraction of dissolved organic matter (SPE-DOM)

For artificial seawater samples (purified artificial seawater, medium 3), about 900 mL medium (six serum bottles, 156 mL each) were pooled at four different time points (blank, t_0_, t_1_, t_2_). The blank was taken directly after medium preparation without inoculation and inoculated samples were taken at different time points of incubation: immediately after inoculation (t_0_), after 1 week (t_1_) and after 3 weeks (t_2_). Serum bottles of natural seawater samples were not pooled and only the time points t_0_ and t_2_ were sampled due to the limited amount of natural oligotrophic seawater available. All samples were filtered into 1 L glass bottles using Acrodisc 25 mm syringe filters with a 0.2 μm GHP membrane (Pall Life Sciences, Ann Arbor, Michigan, USA). Samples for DOC measurements were filled in pre-combusted glass vials and analyzed immediately after sampling. Samples for amino acid analysis were taken into pre-combusted amber glass vials and stored at -20°C. Samples for FT-ICR-MS analysis were acidified to pH 2 with 25% HCl (high purity analytical grade, Merck, Darmstadt, Germany) and stored at 4°C until further analysis.

### Measurement of dissolved organic carbon (DOC) and total dissolved nitrogen (TDN)

DOC and TDN were analyzed using a Shimadzu TOC-VCPH total organic carbon analyzer equipped with a TNM-1 total nitrogen measuring unit and an ASI-V autosampler (Shimadzu, Kyoto, Japan). Within the instrument, samples were acidified with 1% v/v 2 mol L^-1^ HCl (high purity analytical grade, Merck, Darmstadt, Germany) and sparged with synthetic carbon-free air for 2 minutes to remove inorganic carbon. Detection limits were 0.5 μmol L^-1^ for DOC and TDN (0.006 mg C L^-1^ and 0.007 mg N L^-1^, respectively). Analytical errors based on the standard deviations for replicated measurements (at least three measurements per sample) were within 5% for DOC and TDN. Analytical precision and accuracy was tested in each run against deep Atlantic seawater reference material and low carbon water provided by the consensus reference materials program (D.A. Hansell, University of Miami, Florida, USA). Procedural blanks, including the filtration step, were obtained with ultrapure water.

### Amino acid analysis

Concentrations of DFAA and total hydrolysable dissolved amino acids (THDAA) were analyzed using high performance liquid chromatography (HPLC) and pre-column-derivatization with ortho-phthaldiadehyde (Sigma, Taufkirchen, Germany) [21–22]. Prior to the analysis of THDAA, HCl (suprapure grade; Merck, Darmstadt, Germany) was added to a final concentration of 1.7 mol L^-1^ and incubated in fire-sealed glass ampules for 1 hour at 155°C under nitrogen atmosphere. The concentration of DCAA was calculated by subtracting the concentration of DFAA from the concentration of THDAA. Detection limits were 5 nmol L^-1^ for THDAA and 2 nmol L^-1^ for DFAA.

### H^13^CO_3_ and ^15^N_2_ labeling experiments

For labeling experiments, clean artificial seawater (medium 2) was prepared as described above, but the medium was prepared under synthetic air atmosphere (20% O_2_ in N_2_; H_2_O < 3 ppm-mol, C_n_H_m_ < 0.1 ppm-mol, CO < 1 ppm-mol, CO_2_ < 1 ppm-mol; Air Liquide, Krefeld, Germany). From each 156 mL serum bottle, which was completely filled with medium, 5 mL medium were exchanged with 5 mL ^15^N_2_ gas and 300 μL of 1 mol L^-1^ NaH^13^CO_3_ were added. After inoculation with *Pseudovibrio* sp. FO-BEG1, samples were incubated without shaking at 28°C for 12 hours, 3 and 5 days. After incubation, the bacteria were filtered onto pre-combusted Glass Microfibre Filters (GF/F, Whatman, GE Healthcare, Dassel, Germany), frozen for 12 hours and dehydrated in an HCl desiccator. Filters were folded into tin cups and flash combusted (1050°C) within the mass spectrometer to release N_2_ and CO_2_. For analysis, we used an elemental analyzer (with autosampler) coupled to a Delta Plus Advantage mass spectrometer isotope-ratio monitoring mass spectrometer (ThermoFinnigan, Bremen, Germany). To calculate the amount of label in the cells the percentage of label added was taken into account.

### Electrospray ionization Fourier transform ion cyclotron resonance mass spectrometry (ESI FT-ICR-MS)

Prior to ESI FT-ICR-MS, DOM was desalted and concentrated via solid phase extraction (SPE), using Agilent Bond Elut PPL cartridges, following the procedure by Dittmar *et al*. [16]. In brief, approximately 700 mL of artificial seawater medium and approximately 200 mL of natural seawater medium were filtered and acidified (pH 2) and passed via gravity through a cartridge containing 1 g of PPL adsorber. Prior to use, the adsorbers were soaked in methanol (ULC/MS grade, Biosolve, Valkenswaard, The Netherlands) over night, and rinsed with three cartridge volumes (each ca. 6 mL) of methanol and 0.01 mol L^-1^ HCl (high purity analytical grade, Merck, Darmstadt, Germany), each. After adsorption of DOM, the cartridges were rinsed with three cartridge volumes of 0.01 mol L^-1^ HCl, dried under a stream of argon, and solid-phase extractable DOM (SPE-DOM) eluted with 6 mL of methanol into pre-combusted amber glass vials. The samples were stored at -20°C until analysis. For ESI FT-ICR-MS analysis, SPE-DOM extracts were diluted 1:1 with ultrapure water to yield DOC concentrations of 10 mg C L^-1^ (0.83 mmol C L^-1^) for natural seawater and 2 mg C L^-1^ (0.17 mmol C L^-1^) for artificial seawater, respectively (calculated from DOC concentrations in extracts). Due to the extremely low DOC concentration in the artificial seawater samples, higher concentrations were not achievable. Diluted samples were analyzed on a solariX Fourier transform ion cyclotron resonance mass spectrometer (FT-ICR-MS; Bruker Daltonik GmbH, Bremen, Germany) equipped with a 15 Tesla superconducting magnet (Bruker Biospin, Wissembourg, France). Samples were directly infused at a flow rate of 2 μL min^-1^ into an electrospray source (ESI; Apollo II ion source, Bruker Daltonik GmbH, Bremen, Germany) with the capillary voltage set to 4 kV in negative mode and 4.5 kV in positive mode. Ions were accumulated in the hexapole for 0.3 seconds prior to transfer into the ICR cell. Data acquisition was done with broadband scans using 4 megaword data sets and a scanning range of 150–2000 Da. The instrument was first externally calibrated with arginine clusters and then internally calibrated with an in-house marine deep sea DOM reference sample from the deep North Pacific (>100 known C_x_H_y_O_z_ molecular formulas). Before each sample set, blank checks with methanol/ultrapure water 1:1 were measured. For one mass spectrum, 500 broadband scans were accumulated and then internally calibrated with at least 20 different compounds covering the relevant mass range (series of C_x_H_y_O_z_ compounds from 281 to 621 Da). The internal calibration yielded a mass accuracy better than 0.1 ppm (difference between accurate and measured molecular mass). For each detected mass, the molecular formulas were calculated in the mass range between 150 and 850 Da by applying the following restrictions: ^12^C_1-130_^1^H_1-200_O_1-50_^14^N_0-4_S_0-2_P_0-2_ (considering also ^23^Na clusters in ESI positive mode). Assignment of molecular formulas was done with the Bruker software DataAnalysis 4.0 SP 4 using the criteria described by Koch *et al*. [23]. Only compounds with a signal-to-noise ratio of 3 and higher were used for further analysis. The achieved resolving power (full width half maximum) was on average 480,000 at m/z 400.

Each sample was analyzed in triplicate and only masses that were detected in all triplicates were considered for molecular formula assignments. Relative peak heights were then calculated by normalization to the twenty most abundant peaks in each spectrum. The relative intensities of peaks with assigned molecular formulas were used to semi-quantitatively assess changes in DOM concentrations in the incubation experiments. Changes in relative peak intensities between different sampling time points were only considered significant if p < 0.05 when applying a two sided and paired Student’s t-test with Benjamini-Hochberg correction after confirming normal distribution using Shapiro-Wilk’s test of normality. The normalized peak heights were then used to semi-quantitatively assess changes in DOM composition in artificial and natural oligotrophic seawater experiments (tables with FT-ICR-MS data available here: http://doi.pangaea.de/10.1594/PANGAEA.841837). FT-ICR-MS is not a quantitative tool for DOM analysis due to the lack of standards and differences in ionization efficiencies for different compounds. However, this method yields reproducible results because the variability of relative peak heights is low under similar analytical conditions [24].

For known compounds, however, standard addition can be performed and concentrations can be calculated. To quantify the amount of polyethylene glycol (PEG) contamination in the artificial seawater medium an authentic PEG standard (octa-ethylene glycol, C_16_H_34_O_9_, ≥ 99% oligomer purity, 370 g mol^-1^, Sigma, Taufkirchen, Germany) was sequentially added. PEG was added to the medium in concentrations of 1 to 3.6 nmol L^-1^ and FT-ICR mass spectra were recorded in ESI positive mode. The amount of PEG in the samples was calculated from a linear calibration derived from peak heights in the FT-ICR mass spectra.

### Biolog experiment

A substrate respiration test was performed using a Biolog GN2 plate (Hayward, CA, USA) with 95 different substrates. The bacteria were pre-grown in artificial seawater (medium 1) in 1 L bottles (Schott, Mainz, Germany). After 6 days cell numbers were about 10^6^ cells mL^-1^, cells were concentrated by centrifugation for 2 hours at 11,000 × g and 15°C using a J-26XP Beckmann centrifuge (Beckman Coulter GmbH, Krefeld, Germany). All centrifugation tubes were acid washed (0.1 mol L^-1^ HCl) and rinsed with MembraPure water (Optilab-Standard Water System, MembraPure, Bodenheim, Germany). The obtained pellet was suspended in 20 mL of sterile saline solution (402.1 mmol L^-1^ NaCl and 52.1 mmol L^-1^ MgCl_2_ × 6 H_2_O), prepared after combusting all salts (480°C for at least 3 hours). To verify the viability of the cells, 50 μL of the cell suspension was spread on organic-rich agar plates containing 2 g polypeptone, 0.5 g Bacto yeast extract (BD Diagnostics, Heidelberg, Germany), 513.3 mmol L^-1^ NaCl, 24.6 mmol L^-1^ MgCl_2_ × 6 H_2_O, 34 μmol L^-1^ CaCl_2_ × 2 H_2_O, 22.3 μmol L^-1^ Na_2_MoO_4_ × 7 H_2_O, 23.5 μmol L^-1^ CuCl_2_ × 2 H_2_O, 22.2 μmol L^-1^ FeCl_3_ × 6 H_2_O, 15 g agar in 1 L MembraPure water, pH was adjusted to 8 with 1 mol L^-1^ NaOH. The Biolog plate was inoculated with 150 μL of the concentrated cells and incubated at 28°C in the dark in a humidity chamber to prevent excess evaporation. Activity of cells on Biolog plates was checked visually each day for a total of 8 to 14 days.

## Results

### Bacterial cell numbers under oligotrophic conditions

**Fig 1:**
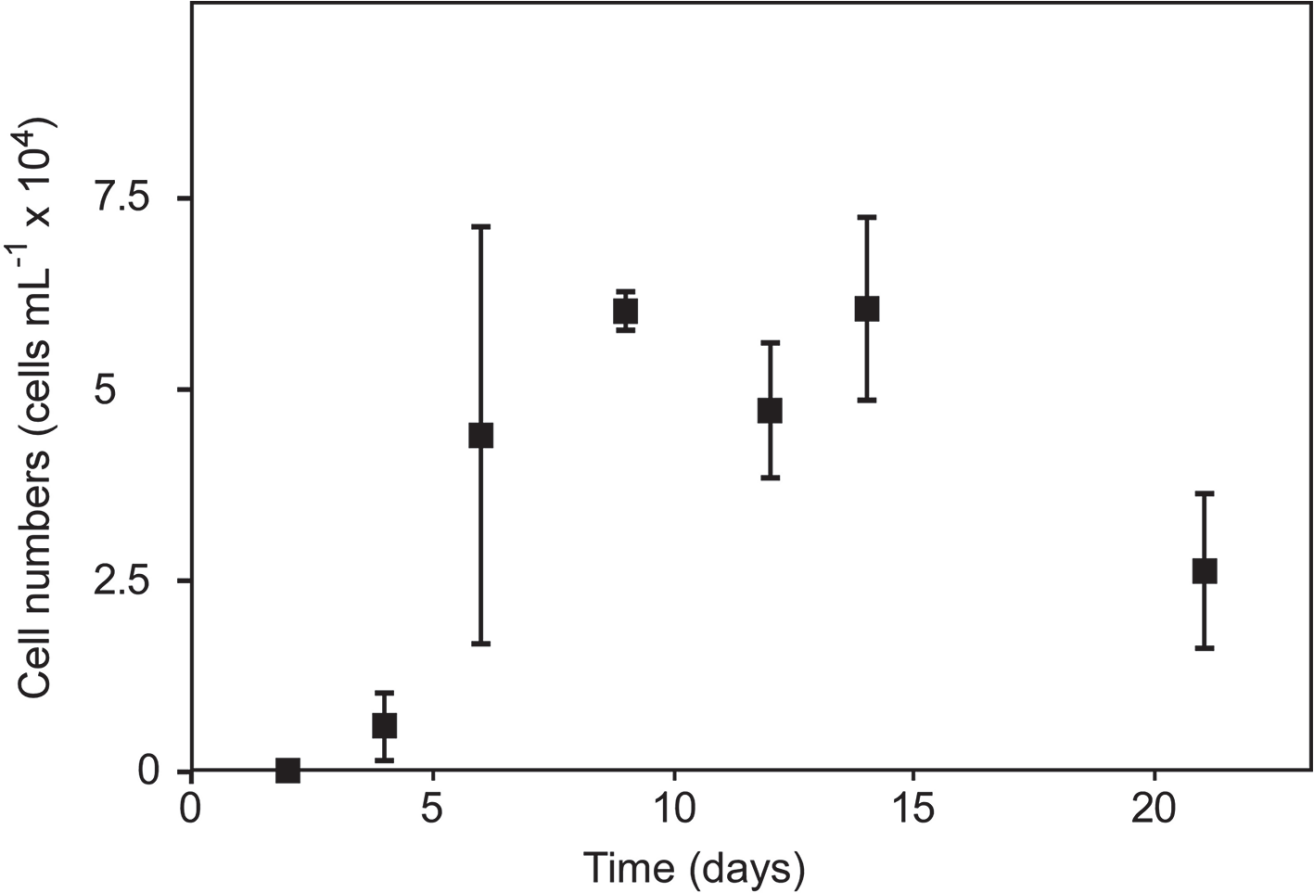
Exemplary growth curve of *Pseudovibrio* sp. FO-BEG1 in clean artificial seawater (medium 2, counted in triplicates). Cell numbers (squares) increased to 6×10^4^ cells mL^-1^ after 1 week of incubation.

The investigated *Pseudovibrio* sp. strain FO-BEG1 was able to multiply in both artificial and natural seawater setups. The initial cell number was calculated to be about 40 cells mL^-1^. Cell numbers in not specially purified artificial seawater medium (artificial seawater, medium 1) were about 3 ×10^5^ cells mL^-1^ (sometimes up to 10^6^ cells mL^-1^) after one week of incubation (Table 2). Interestingly, in clean artificial seawater medium (medium 2), cell numbers increased within one week to only 6×10^4^ cells mL^-1^ (Fig 1 and Table 2). In the FT-ICR-MS experiment using purified artificial seawater (medium 3) cell numbers were even lower with 2×10^4^ cells mL^-1^ (Table 2) at time point t_1_ and did not increase further at t_2_ (one and three weeks after inoculation, respectively). Natural seawater medium used for the FT-ICR-MS experiment contained about 3×10^5^ cells mL^-1^ after three weeks at the end of the incubation (Table 2). Uninoculated controls were checked for all different media used and never contained cells.

**Table 2:** Cell numbers in the different media after 1 and 3 weeks of incubation (initial cell number after inoculation was calculated to be ca. 40 cells mL^-1^).

***Medium***	***Cells mL*** ^-*1*^ ***after 1 week***	***Cells mL*** ^-*1*^ ***after 3 weeks***
Medium 1 (artificial seawater)	3 × 10^5^	2 × 10^5^
Medium 2 (clean artificial seawater)	6 × 10^4^	3 × 10^4^
Medium 3 (purified artificial seawater)	2 × 10^4^	2 × 10^4^
Medium 1 (artificial seawater) + amm.	1 × 10^6^	3 × 10^5^
Medium 1 (artificial seawater) + gluc.	2 × 10^5^	4 × 10^4^
Medium 1 (artificial seawater) + amm. + gluc.	1 × 10^7^	2 × 10^6^
Natural seawater	n.d.	3 × 10^5^

Parallel uninoculated controls were checked for all different media and no cells were observed. (n.d. = not determined; amm. = ammonium; gluc. = glucose)

### Glucose and ammonium addition experiments

The addition of 60 μmol C L^–1^ in form of glucose to artificial seawater (medium 1) increased cell numbers from 3×10^5^ cells mL^-1^ to 28×10^5^ cells mL^–1^ and the addition of 180 μmol C L^–1^ in form of glucose increased the cell numbers to 62×10^5^ cells mL^–1^ (S1 Fig). The separate addition of either glucose or ammonium did only lead to a slight increase in cell numbers, whereas the simultaneous addition of glucose and ammonium increased cell numbers to about 10^7^ cells mL^–1^ (Table 2).

### Biolog experiment

*Pseudovibrio* sp. FO-BEG1 cells pre-grown in artificial seawater medium were used to inoculate a Biolog GN2 microplate containing 95 different growth substrates. Bacterial activity was observed within 8 to14 days on the following substrates: D-raffinose, D-trehalose, turanose, D-gluconic acid, D-glucosaminic acid, D-glucuronic acid, acetic acid, glucuronamide, L-glutamic acid, glycyl-L-glutamic acid, L-serine and glucose-6-phosphate. No activity was observed on the remaining 83 substrates (S1 Table).

### DOC, TDN, DFAA, DCAA and THDAA of dissolved substrates

In the FT-ICR-MS experiment (setup with purified artificial seawater, medium 3) in pure artificial seawater before bottling, the DOC contamination originating from the added main salts was only 1 μmol C L^–1^. With the addition of NaHCO_3_, K_2_HPO_4_, NaOH and trace elements and during the bottling of the medium, the concentration of DOC increased to 4.4 μmol C L^–1^ (Table 1). The TDN concentration was below the limit of detection (<0.5 μmol N L^–1^) in the sterile artificial seawater medium before and after bottling (Table 1). Inoculation did not introduce further measurable DOC or TDN to the medium (t_0_). After one (t_1_) and three weeks (t_2_) of incubation, the concentrations of DOC ranged between 4.3 and 4.8 μmol C L^–1^, which is in the range of the precision of the DOC analyzer (0.5 μmol C L^–1^) and stayed below detection limit (<0.5 μmol N L^–1^) for TDN (Table 1). In comparison to the artificial seawater medium, the DOC concentration in natural seawater was about 8 times higher (36 μmol L^–1^; Table 1). DOC concentrations in inoculated and unioculated samples were comparable during incubation and did not show drastic changes (Table 1). Similarly, TDN concentrations remained stable at 4 μmol N L^–1^ in natural seawater during the incubation (Table 1).

In contrast to DOC and TDN, the amount of DFAA decreased in the artificial seawater cultures within the first week of growth (from t_0_ to t_1_). This decrease represents a DFAA uptake of 0.06 μmol C L^–1^ and 0.02 μmol N L^–1^ (Table 1). After t_1_, the concentrations of DFAA remained constant. The most abundant amino acids were serine, glycine and alanine, which together accounted for 0.05 μmol C L^–1^. The decrease in DFAA in artificial seawater medium (from t_0_ to t_1_) was concurrently observed with an increase of DCAA by the same amount. During the stationary growth phase (from t_1_ to t_2_) the DCAA concentrations decreased again. In contrast to the artificial seawater incubations, concentrations of THDAA did not change during incubation in natural seawater and ranged around 0.1 μmol C L^–1^ and 0.02 μmol N L^–1^ (Table 1).

### Nitrogen and carbon dioxide fixation

The incorporation of labeled nitrogen (^15^N_2_) and carbon (NaH^13^CO_3_) into *Pseudovibrio* sp. FO-BEG1 cells growing in pure artificial seawater medium was monitored using isotope-ratio mass spectrometry. Less than 1% of cellular carbon originated from CO_2_ fixation under the oligotrophic growth conditions in artificial seawater. Nitrogen fixation was not detectable (data not shown).

### Composition of the dissolved organic matter

**Fig 2:**
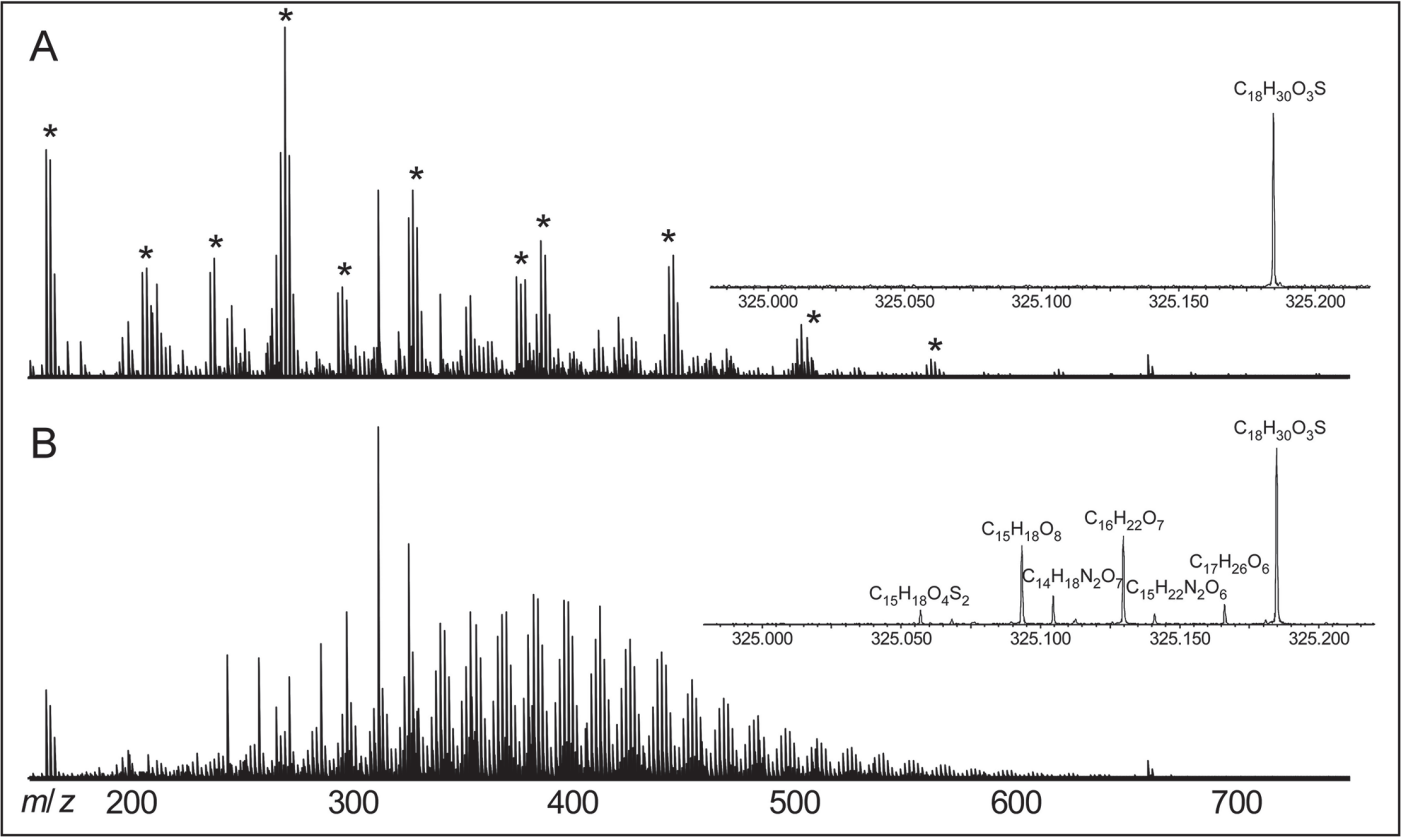
FT-ICR mass spectra (negative electrospray ionization) from the time points t_0_ of (A) artificial seawater and (B) natural seawater. Asterisks indicate inorganic trace impurities. Inserts show a zoom into the region of mass 325.0 to 325.2 Da. In the negative mode inorganic contaminations suppressed the ionization of organic substances present in low amounts in the artificial seawater.

In the natural seawater samples, 6586 molecular formulas were identified in ESI negative mode (Table 3). In the artificial seawater medium, organic molecules were suppressed by inorganic trace impurities in ESI negative mode (Fig 2A). In ESI positive mode, however, 165 molecular formulas were identified in the artificial seawater medium (Table 3). Many of these compounds belonged to two homologous series of intense peaks with distances of 44.02567 Da (C_2_H_4_O_1_) corresponding to polyethylene glycol (PEG) oligomers (S2A Fig). This trace contamination of PEG had been introduced by the inorganic constituents of the artificial seawater, because PEG was detected neither in the blanks nor in natural seawater. PEG is potentially an organic substrate in our incubation experiments, and it can be quantified via the standard addition method. The amount of PEG contamination was quantified by adding defined amounts of a PEG standard to the artificial seawater samples (S2B and S2C Fig). The total amount of PEG in the artificial seawater sample was then calculated to account for 3 nmol C L^–1^, which is three orders of magnitude below the measured DOC concentration in the artificial seawater medium (4.4 μmol C L^–1^). Thus, other compounds than PEG contributed more significantly to DOC in the artificial seawater medium.

**Table 3:** Summary of FT-ICR-MS results: Numbers of assigned formulas, significantly (p < 0.05) changing and decreasing peaks (N-containing compounds were subset of the overall decreasing compounds) in artificial and natural seawater.

***seawater***	***Assigned formulas***	***Significantly changing molecules***		***Significantly decreasing molecules***	***N-containing significantly decreasing molecules***	
Artificial	165		121	109		56
Natural	6586		89	15		6

**Fig 3:**
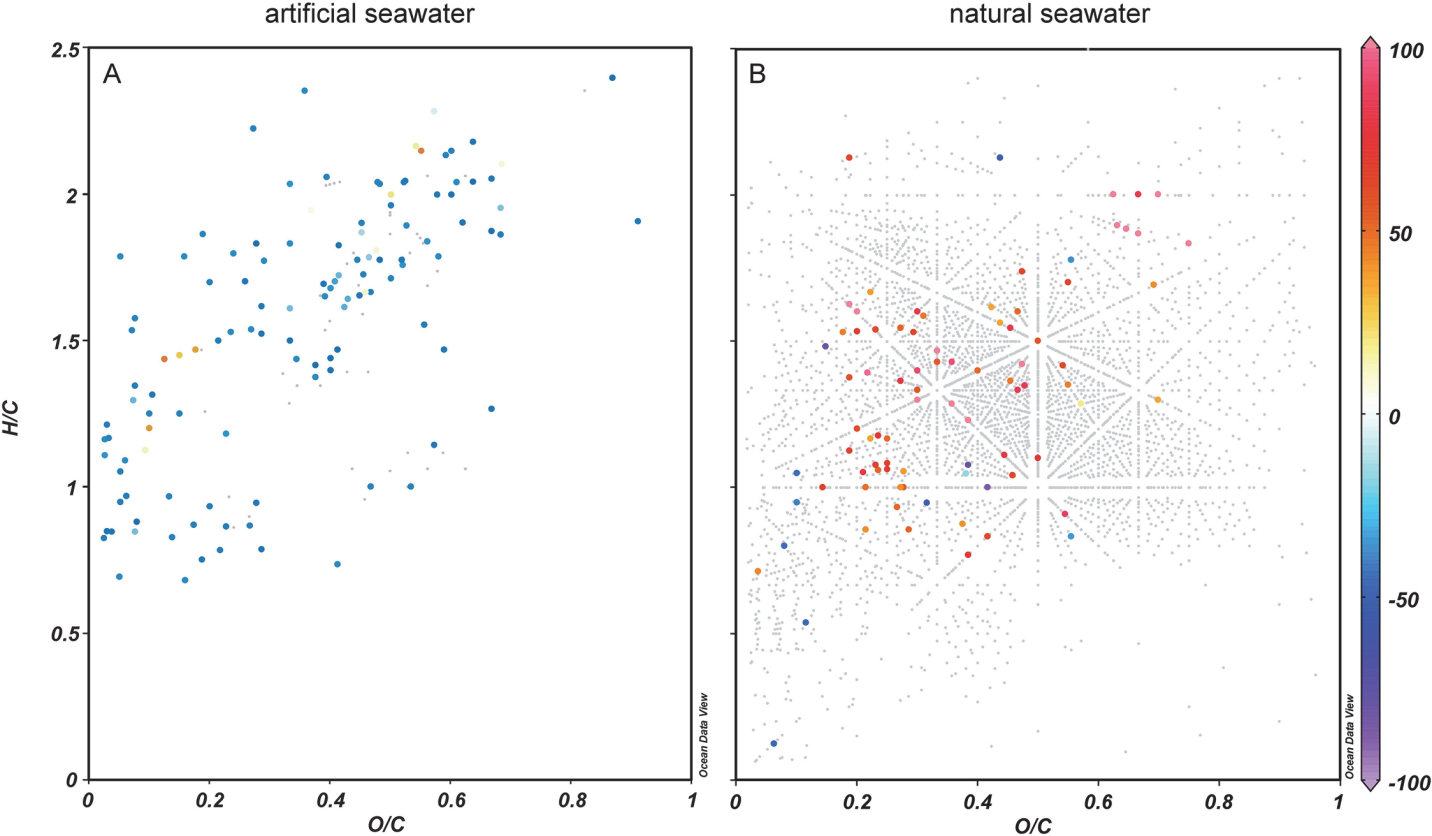
Van Krevelen diagrams (O/C, oxygen to carbon ratio and H/C, hydrogen to carbon ratio of the individual molecular formulas detected) showing the change in DOM composition from t_0_ to t_2_ in artificial (A) and natural seawater (B). Changes in normalized relative intensities (%) are color-coded showing a decrease in blue, an increase in red and no significant change during incubation in grey. In both, artificial and natural seawater, different groups of compounds decreased significantly (p < 0.05) during incubation.

The molecular composition of DOM in the natural seawater medium was very similar to reported ESI negative FT-ICR-MS results of DOM from other oceanic regions [3; 23; 25]. For data illustration, we used van Krevelen diagrams and plotted only the compounds with FT-ICR-MS signal intensities that significantly changed in the course of the incubation. In van Krevelen diagrams, organic compounds are plotted based on their oxygen to carbon (O/C) and hydrogen to carbon (H/C) ratios. During the 3 weeks incubation in artificial seawater (from t_0_ to t_2_) the relative intensities of 121 compounds significantly changed during incubation, of which 109 decreased (Table 3). Compounds with an O/C ≤ 0.4 and H/C ≤ 1.5 and compounds with an O/C ≤ 0.8 and H/C ≥1.25 decreased, while the relative intensity of only few compounds increased (Fig 3A). 56 of the decreasing compounds had molecular formulas containing nitrogen (Table 3). No significant difference was found between the procedural blank of the artificial seawater and t_0_ (before growth). Also, none of the molecular formulas changed in their FT-ICR-MS signal intensities between t_1_ and t_2_ (after growth). The lack of statistically significant differences between these groups of samples and the presence of significant differences between t_0_ and t_2_ confirms the meaningfulness of our choice of comparing only the samples collected at t_0_ and t_2_.

During the incubation with natural seawater, 89 peaks significantly changed, of which 74 increased and only 15 decreased. Six of the decreasing peaks contained nitrogen in the molecular formula (Table 3). Major decreases in incubations with natural seawater during growth (from t_0_ to t_2_) did not occur in a specific group of compounds, but were in general compounds with an O/C ≤ 0.6 and H/C ≥ 0.5 (Fig 3B).

## Discussion

In the present study, the heterotrophic, facultatively oligotrophic bacterium *Pseudovibrio* sp. FO-BEG1 was grown either in ultra-pure artificial seawater medium or in natural oligotrophic seawater (South Pacific Ocean, surface water) representing typical marine DOM. The bacterial growth was sustained by very low amounts of DOC in both incubation setups. However, growth in artificial seawater with only 0.053 mg C L^–1^ (4.4 μmol C L^–1^) shows that bacterial growth can be sustained by even lower concentration than present in most natural oligotrophic seawaters, which typically contain around 1 mg C L^–1^ (83 μmol C L^–1^) [1–2].

### Growth and substrate use in artificial seawater

During growth in purified artificial seawater (medium 3) under extremely oligotrophic conditions, *Pseudovibrio* sp. FO-BEG1 multiplied from about 40 cells mL^–1^ to 2×10^4^ cells mL^–1^ within the first week, even though the overall amount of DOC did not decrease measurably (Table 1). Thus, the amount of compounds that was consumed was within our analytical uncertainty. At the low DOC concentration of the artificial seawater our analytical precision is within approximately 0.5 μmol C L^–1^. Based on the increase in cell numbers when grown after addition of glucose and ammonium, we calculated that about 1 to 3 μmol C L^–1^ would be needed as a carbon and energy source to form the 2×10^4^ cells mL^–1^ after 1 week of growth in artificial seawater, but because of the constant DOC concentration in our experiment, consumption of > 0.5 μmol C L^–1^ is unlikely. The C-need of 1 to 3 μmol C L^–1^ was calculated from cells grown under eutrophic conditions, this value might overestimate the actual C-requirement under oligotrophic conditions. The uptake of amino acids alone could not sustain bacterial growth because the initial amino acid concentration (equivalent to 0.13 μmol C L^–1^, see Table 1) was already lower than the required 1 μmol C L^–1^. In drinking water research the amount of AOC is calculated from the number of cells grown on the DOC available. It is known that in drinking water 0.083 μmol (1 μg C) in form of acetate are needed to form 10^6^ cells [7; 26]. Using this value we would require 0.3 μmol C L^–1^ in form of acetate for 2×10^4^ cells mL^–1^ (and 1.3 μmol C L^–1^ for 10^5^ cells mL^–1^) which is lower than our calculated amount of carbon needed. However, this amount would be within our uncertainty of the analysis (0.5 μmol C L^–1^) and thus would not have been detected.

During the initial growth phase, we found a decrease in DFAA concentration concurrently with an increase in DCAA, whereas the overall amino acid concentration (DFAA + DCAA) did not decrease (Table 1). Within this growth phase, a recycling might have occurred where DFAA might have been used, which lead in turn to an increase in the DCAA concentration, but no overall decrease in the amino acid concentration. The ability to secrete amino acids during growth and the ability to recycle the molecules present in the growth medium was recently underlined by a metabolic study conducted on the same bacterial strain investigated in our study [13]. In the present study, we could actually measure a slight decrease of total amino acids during the stationary growth phase. This may suggest that amino acids were likely used as substrate for maintaining non-growing cells.

Trace impurities of organic compounds were present in the artificial seawater at detectable concentrations. The compositional analysis of DOM in the artificial seawater with ESI FT-ICR-MS revealed a decrease of nitrogen-containing compounds during incubation (Table 3) underlining the ability of the strain to meet its energy, carbon and nitrogen demand by incorporating a variety of different compounds. Using this method we cannot absolutely quantify the amount of carbon that corresponded to this decrease. In addition, a compound with a decreasing peak in the mass spectrum is not necessarily completely oxidized to CO_2_. Thus, we cannot ultimately state that the use of these substrates alone explains the observed growth. Nevertheless, the fact that about half of the decreasing compounds contained nitrogen suggests that these trace impurities at least served as nitrogen source. This is in agreement with the observation that N_2_ fixation by *Pseudovibrio* sp. FO-BEG1 under oligotrophic conditions in artificial seawater was not detectable. According to Sleighter and Hatcher [27] and Kim *et al*. [28] compounds with an O/C ≤ 0.4 and H/C ≤1.5 most likely resemble condensed hydrocarbons and aromatic compounds, whereas compounds with an O/C ≤ 0.8 and H/C ≥ 1.25 most likely resemble aliphatic amines/amides, proteins and amino sugars/carbohydrates. Most of these compounds contain nitrogen, such as the decreasing compounds. These substrates would therefore likely be suitable substrates for the bacteria under oligotrophic conditions and could explain the activity and growth of the bacteria.

Among the identified compounds, we detected two series of polyethylene glycol oligomers. The total amount of PEG contamination was about 1000-fold less than the total DOC concentration and a decrease in PEG was not observed in the course of the incubation. Hence, we conclude that PEG was not a significant C source in the artificial seawater incubation.

### Growth and substrate use in natural seawater

The overall concentration of amino acids in the natural seawater was low initially and did not decrease during the incubation. Thus, amino acids alone did not sustain growth or survival of cells, which was expected since we used aged natural seawater where concentrations of labile organic matter should be low. The compositional analysis of DOM showed a significant decrease of only 15 compounds, of which six contained nitrogen (Table 3). Notably, during growth of *Pseudovibrio* sp. FO-BEG1 in natural seawater, we observed a decrease of compounds belonging to diverse groups of substances instead of a decrease of specific compound groups, i.e. the dynamic compounds appeared in vastly different regions of the van Krevelen plot (Fig 3B). We found only very few significantly decreasing compounds during the incubation with natural seawater. Either these few compounds were sufficient for growth or statistically not significantly decreasing compounds were used as growth substrate.

### The use of multiple substrates under oligotrophic conditions

A vast variety of compounds can be metabolized by bacteria facing oligotrophic conditions to sustain their growth. For example *E*. *coli*, pre-adapted to carbon-limiting conditions, is able to utilize many different substrates [29]. However, it is not known how many different types of compounds may be used simultaneously by bacteria, because each compound is present in low concentration. It is well known that only a fraction of the DOC present is utilized in freshwater systems including drinking water, the so called AOC. With increasing AOC concentrations, higher cell numbers were observed but the composition of the AOC was not studied in detail [30]. Using ESI FT-ICR-MS we could show that, under our incubation conditions, a diverse suite of molecules can be used simultaneously. Among them were different types of organic compounds, with vastly different oxygen to carbon (O/C) and hydrogen to carbon (H/C) ratios, as well as nitrogen contents. Furthermore, the bacteria were able to utilize different compounds under the two different oligotrophic growth conditions with artificial and natural seawater. The high catabolic versatility underlined by the FT-ICR-MS data could be partially confirmed by the Biolog experiment, where also substrates not added to the initial medium could be utilized by oligotrophically pre-grown cells. It is important to point out that Biolog plates only test the use of substrates which are typically added to cultivation media, but these compounds are not necessarily the same substrates which are present in natural environments. The main aim of the performed Biolog experiment was to show that the bacteria are prepared to utilize compounds that they were not exposed to. This shows the metabolic versatility of the bacteria and their ability to use several different substrates. Biolog experiments are useful to show the potential to use a substrate. Our incubation experiments show for the first time under oligotrophic conditions the actual simultaneous decrease of multiple compounds present in original concentrations.

Our data support the hypothesis that the simultaneous use of different compounds may enable bacteria to grow on very low concentrations of each of the different substrates [31–32], if a substrate does not repress enzymes needed for the degradation of another less efficient one [33]. This strategy could enable bacteria to survive in habitats with a low and fluctuating supply of nutrients, as can be found in the ocean. This strategy was shown for *E*. *coli* before [29] and we could confirm it for the metabolically versatile *Pseudovibrio* strain investigated here. It is likely, that this strategy also applies for other heterotrophic bacteria in the marine environment. Moreover, we showed that even under extremely oligotrophic conditions, the isolated bacteria were not in a resting state, but showed a moderate growth, even though nitrogen, carbon and energy sources were limiting factors at the same time. These data provide evidence for redefining the lowest limits of nutrients necessary to sustain bacterial growth. Furthermore, we were able to show that ESI FT-ICR-MS is a powerful tool to investigate bacterial substrate utilization under low-nutrient conditions as it detects a broad range of different organic molecules.

Our results illustrate that the concentration of bulk DOC alone cannot be used as a sole criterion to define oligotrophy. Even at the relatively high DOC concentrations in the ocean, bacteria appear limited by the availability of organic substrate, which is related to its molecular composition [34]. On the other hand, even at comparably low bulk DOC concentrations, heterotrophic life can be supported by the abundance of few highly assimilable compounds. Apparently, at least certain bacteria are able to grow on trace amounts of DOM present in ultra-pure artificial seawater or in ultra-oligotrophic natural seawater using a diverse pool of organic compounds. This is consistent with observations from drinking water research [7; 26].

## Supporting Information

S1 Fig Addition of 60 μmol mL^-1^ carbon in form of glucose leads to an increase from 3×10^5^ cells mL^-1^ to 2810^5^ cells mL^–1^ and the addition of 180 μmol L^–1^ carbon increased the cell numbers to 62×10^5^ cells mL^–1^. (TIF)

S2 Fig Trace impurities of polyethylene glycol (PEG) in artificial seawater: (A) FT-ICR mass spectra from an artificial seawater extract (ESI positive mode) showing high peaks of PEG impurities with intervals of 44.02567 Da. **(B)** FT-ICR mass spectra after standard addition of a selected PEG standard compound in the range of 0 to 3.6 nmol L^-1^ to the artificial seawater extract. **(C)** Linear calibration curve of the selected PEG standard compound after addition to the sample. The intercept of the linear function is the actual concentration of PEG in the sample.
(TIF)

S1 Table Substrates tested with the Biolog plate. (DOCX)
