# Supplementary figures and images for: Substrate Use of Pseudovibrio sp. Growing in Ultra-Oligotrophic Seawater
Source: PLoS One. 2015 Mar 31;10(3):e0121675. doi: 10.1371/journal.pone.0121675 (PMC4380363; doi:10.1371/journal.pone.0121675)

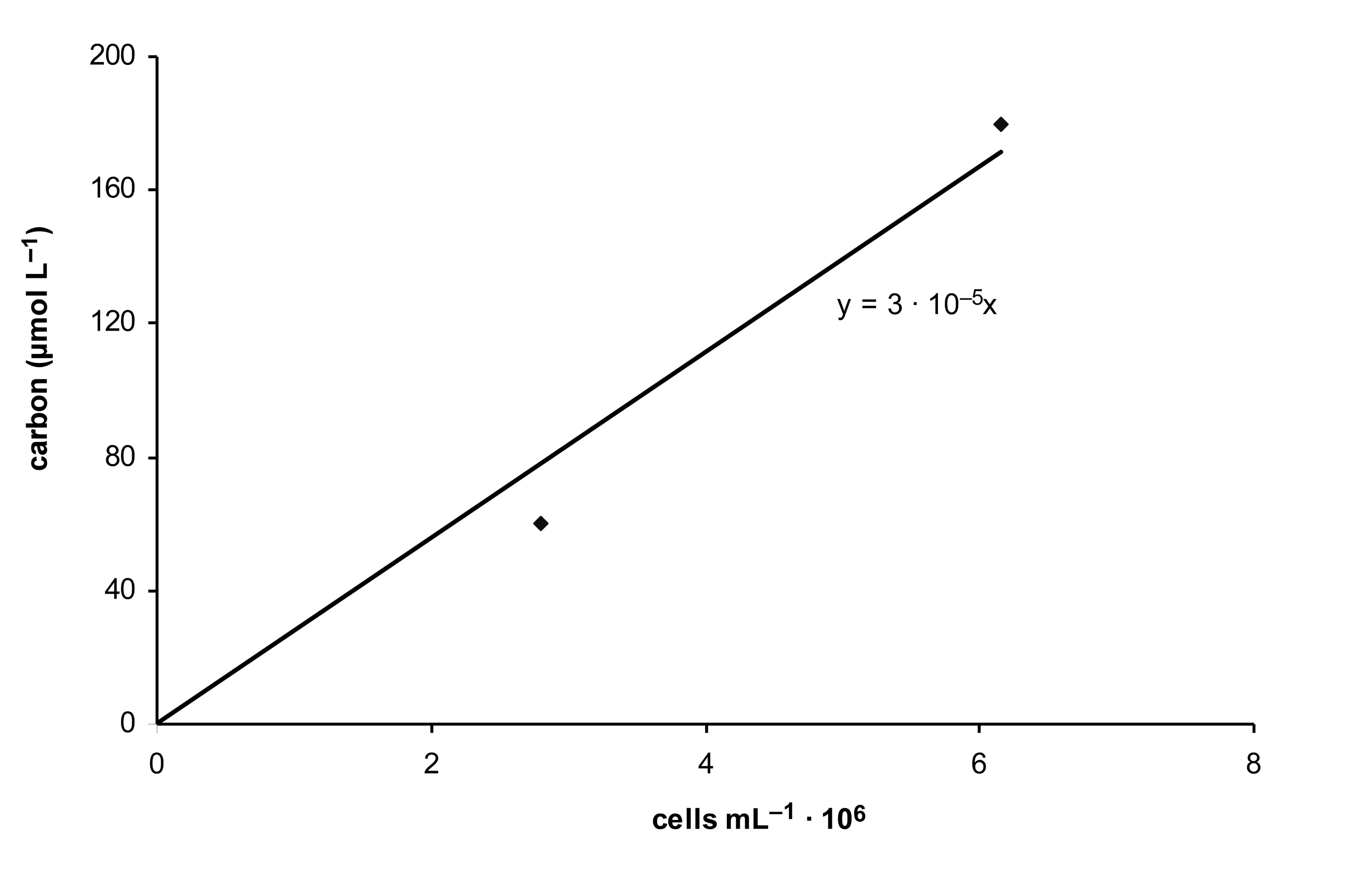

Supplement: S1 Fig — (TIF) [file pone.0121675.s001.tif]

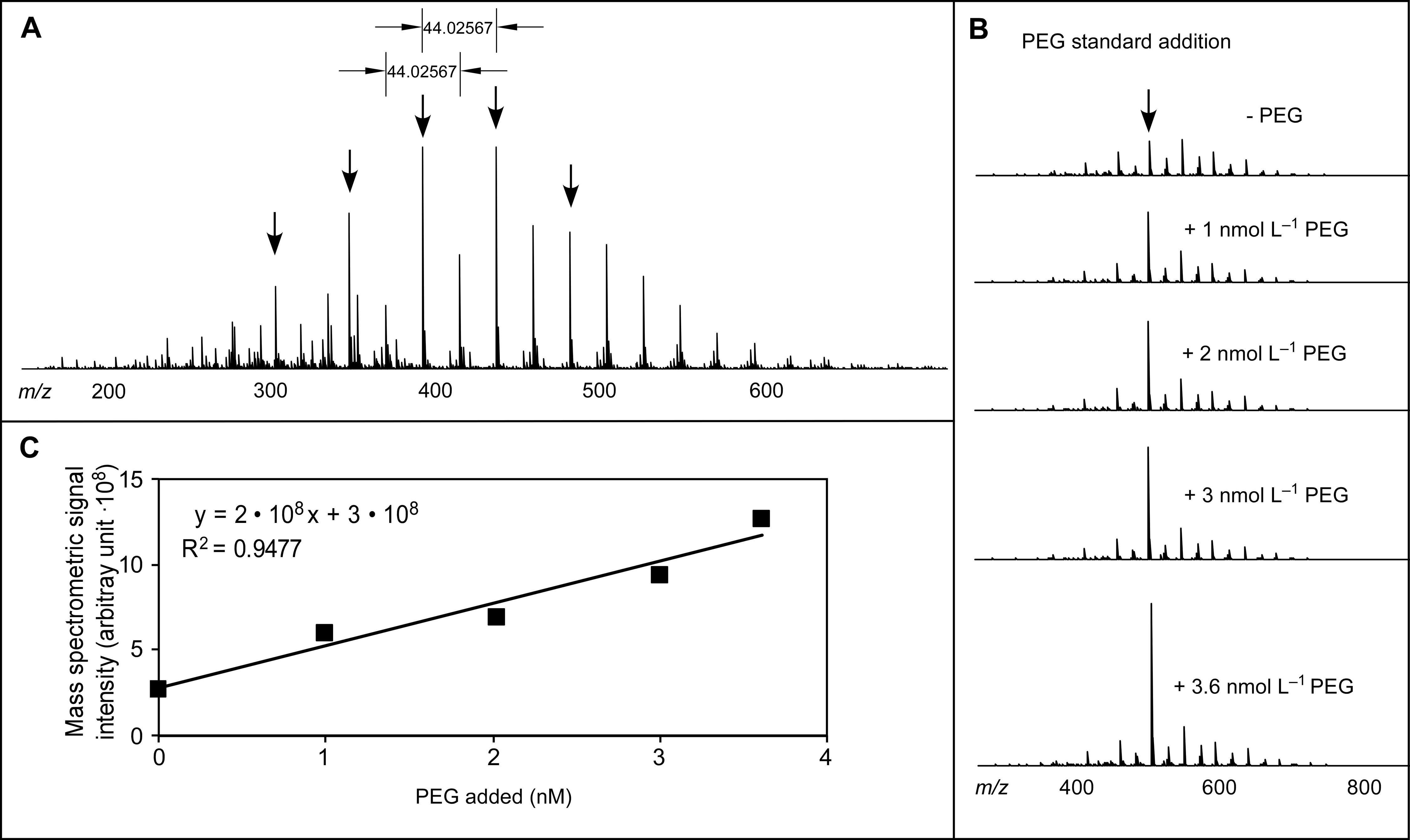

Supplement: S2 Fig — (B) FT-ICR mass spectra after standard addition of a selected PEG standard compound in the range of 0 to 3.6 nmol L-1 to the artificial seawater extract. (C) Linear calibration curve of the selected PEG standard compound after addition to the sample. The intercept of the linear function is the actual concentration of PEG in the sample. (TIF) [file pone.0121675.s002.tif]
