# Supplementary material for: Substrate Use of Pseudovibrio sp. Growing in Ultra-Oligotrophic Seawater
Source: PLoS One. 2015 Mar 31;10(3):e0121675. doi: 10.1371/journal.pone.0121675 (PMC4380363; doi:10.1371/journal.pone.0121675)
Supplement: S1 Table — (DOCX) [file pone.0121675.s003.docx]

**Table S1:** Substrates tested with the Biolog^TM^ plate
